# Estrogen and Progesterone hormone receptor expression in oral cavity cancer

**DOI:** 10.4317/medoral.21182

**Published:** 2016-07-31

**Authors:** Martin Grimm, Thorsten Biegner, Peter Teriete, Sebastian Hoefert, Michael Krimmel, Adelheid Munz, Siegmar Reinert

**Affiliations:** 1 MD, DDS, PhD. Department of Oral and Maxillofacial Surgery, University Hospital Tuebingen, Osianderstrasse 2-8, 72076 Tuebingen, Germany; 2 Department of Pathology, University Hospital Tuebingen, Liebermeisterstrasse 8, 72076 Tuebingen, Germany; 3 Cancer Research Center, Sanford-Burnham Medical Research Institute, 10901 North Torrey Pines Road, La Jolla, CA 92037, USA

## Abstract

**Background:**

Recent studies have shown an increase in the incidence of oral squamous cell carcinoma (OSCC) in younger patients. The hypothesis that tumors could be hormonally induced during pregnancy or in young female patients without the well-known risk factors alcohol or tobacco abuse seems to be plausible.

**Material and Methods:**

Estrogen Receptor alpha (ERα) and Progesterone Receptor (PR) expression were analyzed in normal oral mucosa (n=5), oral precursor lesions (simple hyperplasia, n=11; squamous intraepithelial neoplasia, SIN I-III, n=35), and OSCC specimen. OSCCs were stratified in a young female (n=7) study cohort and older patients (n=46). In the young female study cohort three patients (n=3/7) developed OSCC during or shortly after pregnancy. Breast cancer tissues were used as positive control for ERα and PR expression.

**Results:**

ERα expression was found in four oral precursor lesions (squamous intraepithelial neoplasia, SIN I-III, n=4/35, 11%) and in five OSCC specimen (n=5/46, 11%). The five ERα positive OSCC samples were older male patients. All patients within the young female study cohort were negatively stained for both ERα and PR.

**Conclusions:**

ER expression could be regarded as a seldom risk factor for OSCC. PR expression seems to be not relevant for the development of OSCC.

**Key words:**Oral squamous cell carcinoma, estrogen receptor, progesterone receptor, hormone receptor.

## Introduction

Oral squamous cell carcinoma (OSCC) is typically regarded to be a disease that predominantly affects older males (1,2). Intriguingly, recent studies have shown an increase in the incidence of OSCC in young female patients without the well-known causes of OSCC like alcohol and tobacco abuse (2-4). The etiology and pathogenesis of oral cavity cancer in young female patients could be different from those occurring in older patients (3,5). Pregnancy has been shown to be associated with OSCC but there is a paucity of data regarding this etiology as these studies are primarily case reports discussing the challenges that clinicians face in administering treatment that is of maximal benefit to the patient and minimal risk to the fetus (4,6-10).

The hypothesis that tumors could be hormonally induced during pregnancy or in young female patients without the well-known risk factors seems to be plausible. However, it has not yet been determined whether or not a biological predisposition to OSCC exists. In the context of oral cavity carcinogenesis, studies have been shown that hormone receptors, like Estrogen Receptor (ER) and Progesterone Receptor (PR) expression could be regarded as a biological predisposition factor for OSCC (11-15).

Therefore, in our study we compare a series of OSCCs, which were stratified in a young (<45 years (16)) female study cohort and older OSCC patients. In the young female OSCC study cohort three patients developed OSCC during or shortly after pregnancy.

Analysis of the mechanistic basis in OSCC development in the context of a multistep carcinogenetic process through morphologically and clinically detectable precancerous stages (17) may harbour the availability of molecular tools to selectively and experimentally manipulate this multistep process. Therefore, ER and PR expression has been additionally analyzed in squamous intraepithelial neoplasia (SIN) lesions.

## Material and Methods

- Patients and Tumor Specimen 

The records of healthy individuals (normal oral mucosal tissues, n=5), patients with oral precursor lesions (simple hyperplasia, n=11; squamous intraepithelial neoplasia SIN I, n=5; SIN II, n=9; SIN III, severe dysplasia, n=10; SIN III, carcinoma in situ, n=11), and patients with invasive OSCC were retrospectively assessed from January 2009 to November 2014. OSCCs were stratified in a young (<45 years (16)) female (n=7,  Table 1) study cohort and older patients (n=46,  Table 2) (18). In the young female study cohort three patients (n=3/7) developed OSCC during or shortly after pregnancy. The diagnosis of normal oral mucosal tissues, precursor lesions, and invasive squamous cell carcinoma was confirmed by the department of Pathology, University Hospital Tuebingen. The material was archival formalin-fixed, paraffin-embedded tissue from routine histopathological work-ups. Both OSCC study cohorts were negatively assessed for human papillomavirus (HPV) in routine analysis by using fluorescence in-situ hybridization (FISH) testing. The material has been stored with permission of the local ethics committee of the University Hospital Tuebingen (approval number: 562-2013BO2), after informed consent obtained from the patients prior to surgical resection. Tumor blocks of paraffin-embedded tissue were selected by experienced pathologists, based on routine H&E stained sections. Sections from all available tissues underwent histopathological assessment, blinded to the prior histopathology report. Serial tissue sections (2 µm thickness) were cut from formalin-fixed paraffin-embedded (FFPE) blocks on a microtome and mounted from warm water onto adhesive microscope slides. First, we assessed H&E stained sections from each tissue section to differentiate between normal tissue, precursor lesions, tumor cell areas, stromal areas, and infiltrating immune cells. Breast cancer tissues were used as a representative positive control. Oral precursor lesions were classified according to WHO criteria (17). Tumor staging was performed according to the 7th edition of the TNM staging system by the UICC/ AJCC of 2010. Grading of OSCC was defined according to WHO criteria.

**Table 1:**
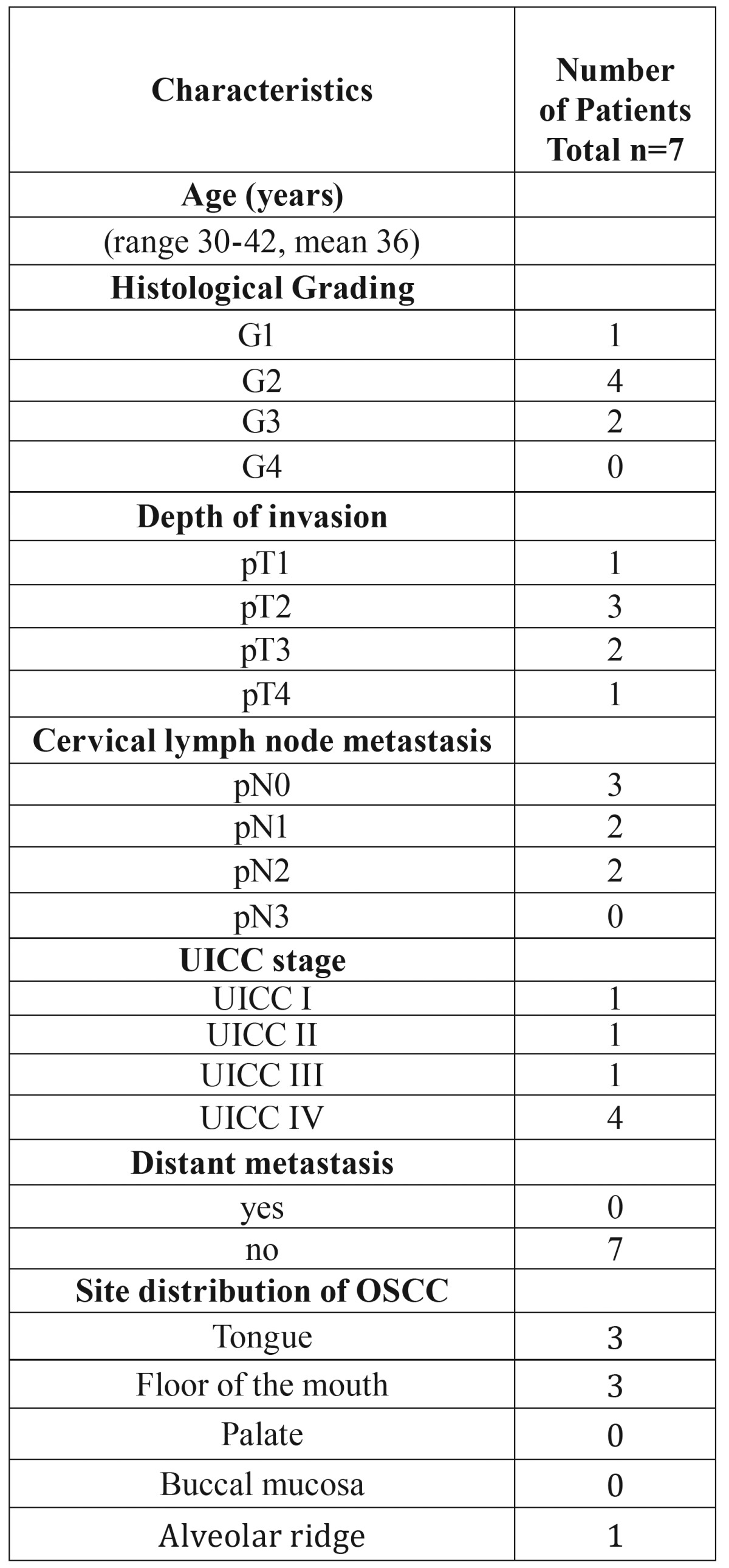
Clinicopathological characteristics of 7 young female patients with OSCC.

**Table 2:**
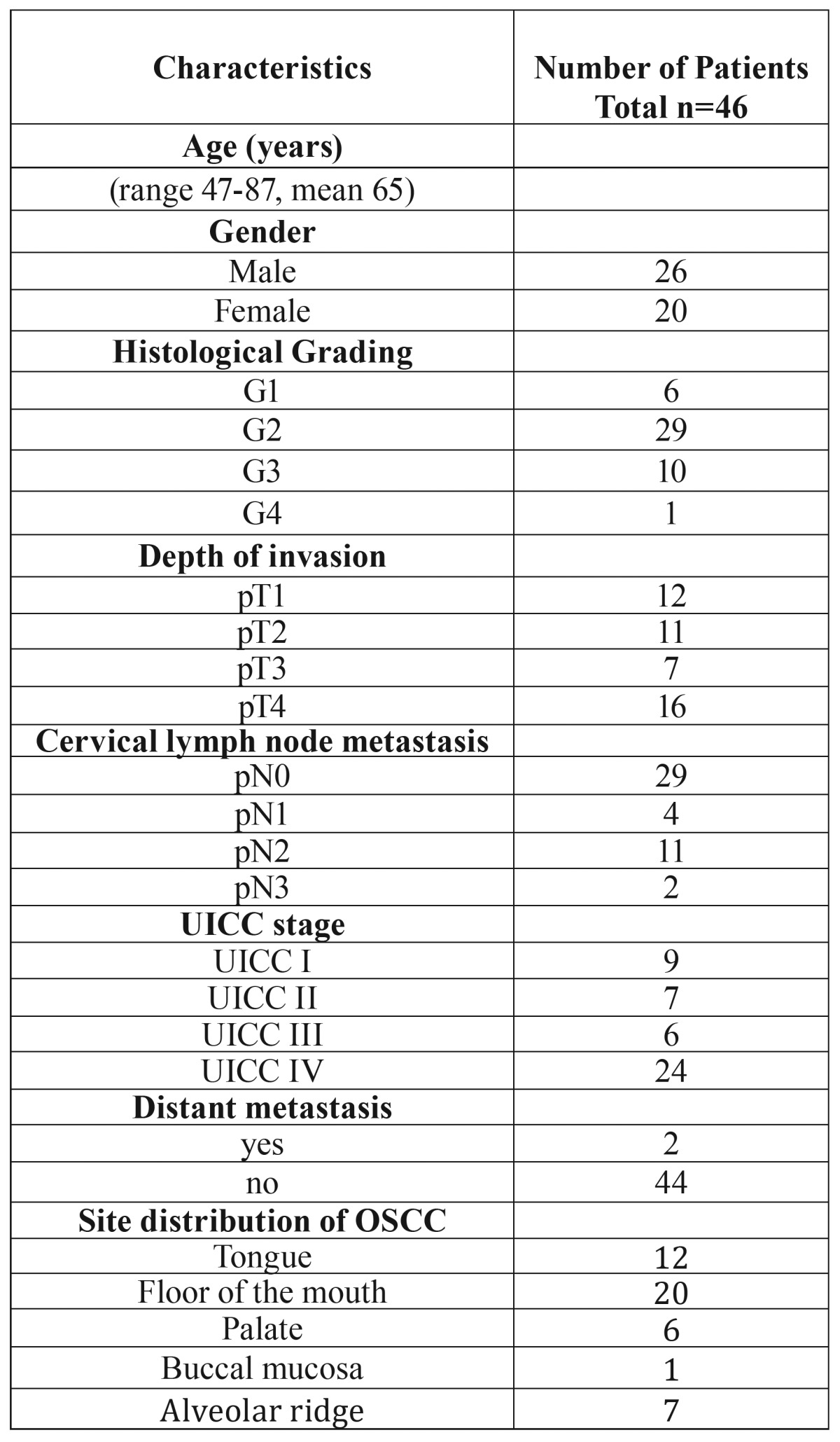
Clinicopathological characteristics of 46 patients with OSCC.

- Staining procedure and quantification of immunohistochemistry

We stained for Estrogen Receptor alpha (ERα, Dako Cytomation, Hamburg, Germany, rabbit mAb, Code M3643, Clone EP1, dilution 1:50), Progesterone Receptor (PR, Dako Cytomation, mouse mAb, Code M3569, Clone PgR 636, dilution 1:50), and mouse/rabbit isotype controls (BD Pharmingen, Heidelberg, Germany) in tissue sections. Staining was performed on serial sections of 2µm thickness, which were deparaffinized in xylene and ethanol and rehydrated in water. Heat induced epitope retrieval (HIER) was performed with either citrate buffer pH 6.0 (Dako, Hamburg, Germany) or EDTA buffer pH 9.0. Endogenous peroxidase activity was quenched with 0.3% hydrogen peroxide. Endogenous biotin activity was blocked using the avidin/biotin blocking kit (Vector Laboratories, Burlingame, CA, USA). After incubation with the primary or rabbit control antibody (BD Pharmingen, Heidelberg, Germany (19) the Dako LSAB2 peroxidase System (Dako, Hamburg) was used. Slides were subsequently incubated for 3-5 minutes in DAB (3,3’-diaminobenzidine, Biogenex) counterstained with haemalaun and mounted with Glycergel (Dako).

Five representative high power fields (1 HPF = 0.237 mm2, original magnification: x200-fold) were analyzed. The extent of the staining, defined as the percentage of positive staining areas of tumor cells in relation to the whole tissue area, was semi-quantitatively scored. A positive result was defined as nuclear staining in ≥1% of tumor cells (20). Two observers blinded to the diagnosis performed scoring on identical sections marked by circling with a water-resistant pencil and finally with diamond-tipped pencil on the opposite side of the microscopic slide. Pictures were analyzed using a Canon camera (Krefeld, Germany). The photographed images were imported into the Microsoft Office Picture Manager.

- Statistical analysis

Statistical analysis was performed with MedCalc Software, Version 15.8 (Mariakerke, Belgium). Descriptive statistics were generated according to case-control status.

## Results

- Expression of ERα and PR in normal mucosa, oral precursor lesions and OSCC

**Figure 1:**
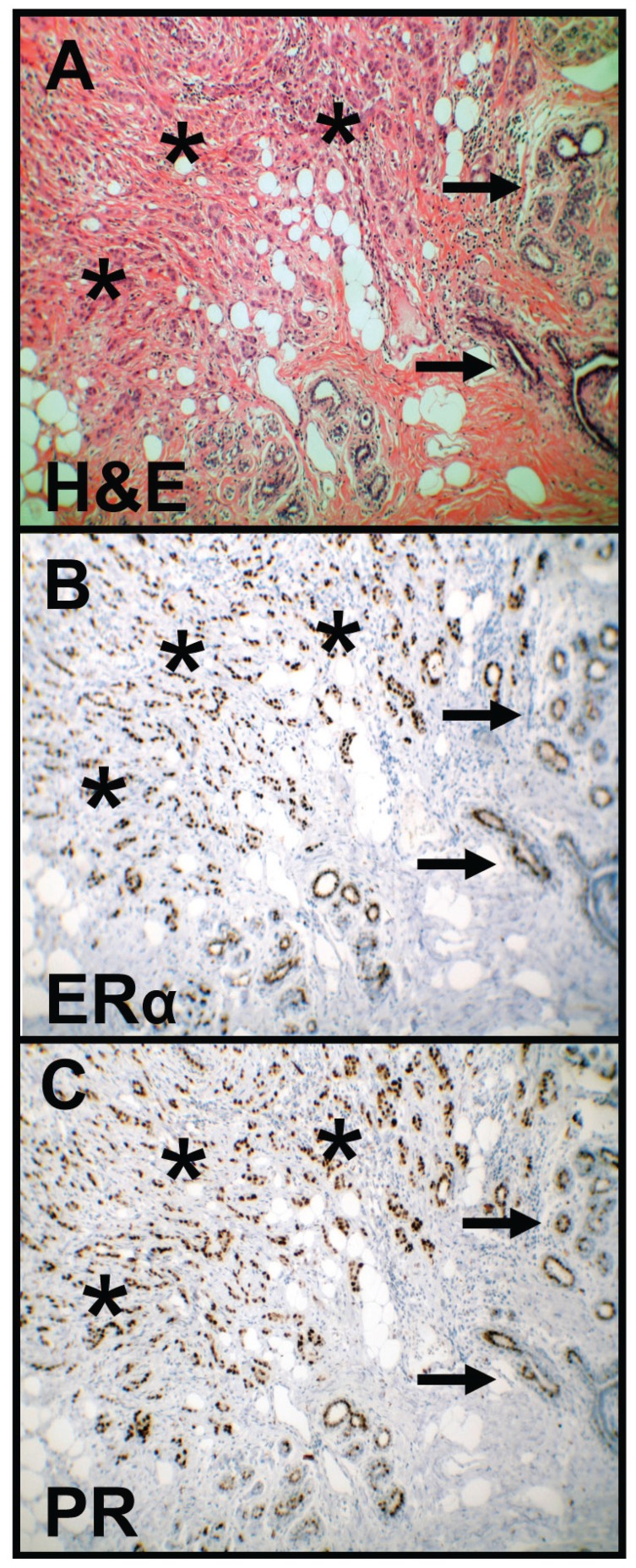
HE staining and immunohistochemical staining of ERα and PR in breast cancer. HE staining (A) shows tumor cells (asterisks) and normal mammary gland tissue (arrows). Immunohistochemical staining shows representative images of positive ERα (B) and PR (C) expression in breast cancer as a positive control (asterisks). Brown chromogen color (3,3’-Diaminobenzidine) indicates positive nuclear staining, the blue color shows the nuclear counterstaining by hematoxylin (original magnification: x100-fold). HE, Haematoxylin and eosin; ERα, Estrogen Receptor alpha; PR, Progesterone Receptor.

**Figure 2:**
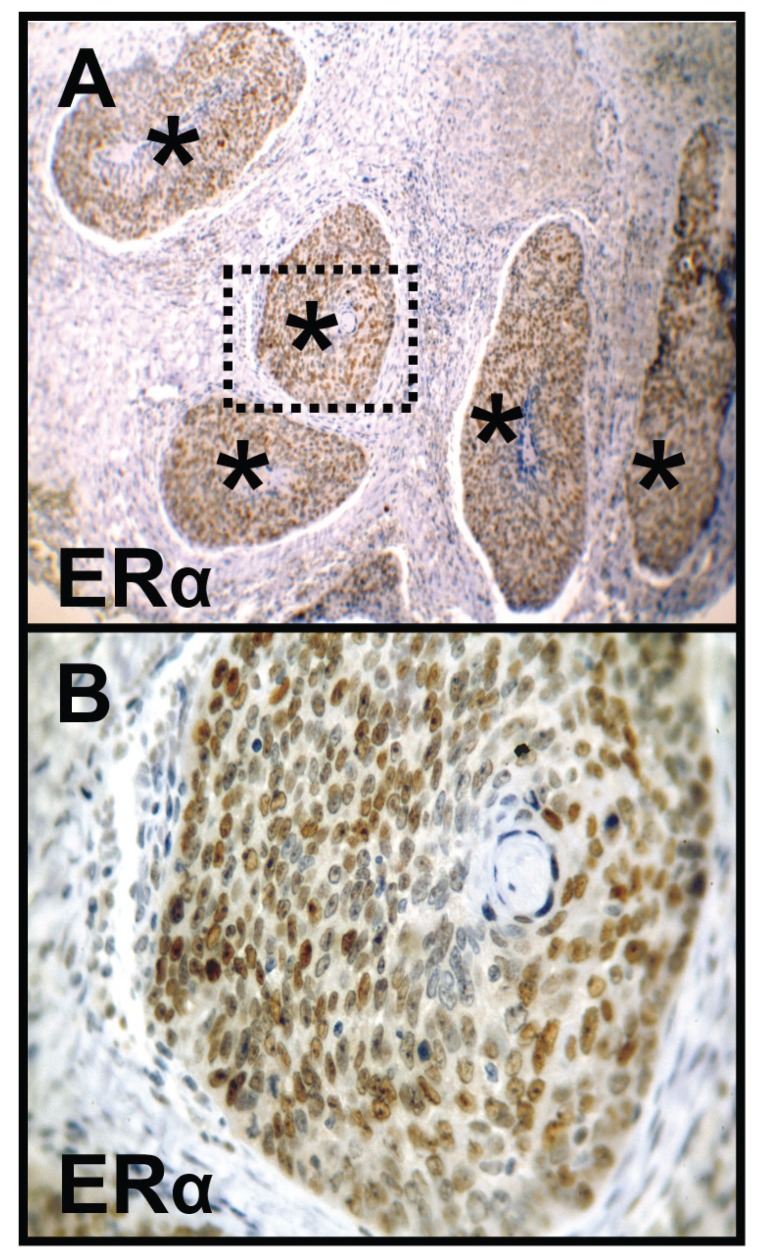
Immunohistochemical staining of ERα in OSCC. Immunohistochemical staining shows representative images of positive ERα expression (A, B) in OSCC (asterisks). Brown chromogen color (3,3’-Diaminobenzidine) indicates positive nuclear staining, the blue color shows the nuclear counterstaining by hematoxylin. The square box demonstrates the area of interest (original magnification: x100-fold, upper panel, A), which is also shown in larger magnification (x400-fold, lower panel, B). ERα, Estrogen Receptor alpha; OSCC, oral squamous cell carcinoma.

Breast cancer tissues were used as a representative positive control for studying ERα and PR expression (Fig. 1). ERα expression was not found in normal oral mucosa (n=0/5) and simple hyperplasia (n=0/11) but in four oral precursor lesions (squamous intraepithelial neoplasia, SIN I-III, n=4/35, 11%) and in five OSCC specimen (n=5/46, 11%, Fig. 2). Positive ERα expression was only found in the older-aged OSCC study cohort. The five positive samples were older male (age: 54-73 years) patients (no female). All patients within the young female study cohort (n=7, including the three OSCC patients, who developed the tumor during or shortly after pregnancy) were negatively stained for ERα.

PR expression was not found in normal oral mucosa (n=0/5), oral precursor lesions (simple hyperplasia, n=0/11; squamous intraepithelial neoplasia, SIN I-III, n=0/35), and OSCC specimen of both study cohorts (older-aged OSCC patients, n=0/46; young female OSCC patients n=0/7).

## Discussion

In-vitro studies have shown ERα expression in OSCC cell lines and tumors. The treatment with tamoxifen significantly inhibits OSCC cell proliferation and invasion (14,15,21-23). Therefore, the usage of tamoxifen for targeted therapies may be useful for hormonally active OSCCs. In our study, we analyzed the ERα subunit as the alpha subunit plays a predominant role in the promotion of cell growth and survival (24). A study of 24 OSCC specimens demonstrated that the frequency of ERα expression was 50% (15). Moreover, a previous study by Chang *et al.* (14) showed that ERα immunoreactivity was observed in 43% of malignant lesions, whereas none of benign lesions showed ERα immunoreactivity. In our survey, by using well established monoclonal antibodies we detected 11% positive OSCC samples, which is much lower than the reported prevalence in the current literature but 11% positive SIN lesions that hasn´t been reported as yet.

The activation status of ERα and the regulatory mechanism of ERα activation in OSCC cells are mostly unknown (14). The results published by Chang *et al.* (14) suggest that ERα activity can be enhanced by focal adhesion kinase (FAK)/Protein kinase B (AKT) signalling, which is critical for promoting cell growth in OSCC cell lines. Moreover, a cross-talk between ER and epidermal growth factor receptor (EGFR) in head and neck squamous cell carcinoma cell lines has been reported (25).

Intriguingly, positive ERα expression was only found in the older-aged OSCC study cohort. The five positive samples were older male patients (no female). All patients within the young female study cohort (including the three OSCC patients, who developed the tumor during or shortly after pregnancy) were negatively stained for both ERα and PR. Therefore, sexual hormone receptor expression could not be regarded as a risk factor for young female OSCC patients or pregnancy. The reason for ERα expression in older male patients remains unclear.

## Conclusions

Based on the results of the present study sexual hormone receptor expression is not associated with young OSCC female patients or pregnancy. ER expression could be regarded as a seldom risk factor for OSCC, whereas PR expression seems to be not relevant for the development of OSCC.

- Abbreviations

SIN, squamous intraepithelial neoplasia; OSCC, oral squamous cell carcinoma; ERα, Estrogen Receptor alpha; PR, Progesterone Receptor.
